# Ultrasound Detection of *Salmonella* Septic Arthritis in a Rheumatoid Arthritis Patient on Anti-TNF Treatment

**DOI:** 10.1177/2324709614532799

**Published:** 2014-04-17

**Authors:** Anupama C. Nandagudi, Stephen Kelly

**Affiliations:** 1 Basildon and Thurrock Hospital NHS Foundation Trust, Basildon, UK; 2 Barts and London NHS Trust, London, UK

**Keywords:** musculoskeletal ultrasound, rheumatoid arthritis, *Salmonella*, septic arthritis

## Abstract

We report a case of *Salmonella* septic arthritis detected by ultrasound in a 40-year-old man with rheumatoid arthritis while he was on anti–tumor necrosis factor-α monoclonal antibody certolizumab. An aspirate of his left elbow joint showed *Salmonella* enteritidis infection that was sensitive to ceftraixone. This was preceded by a brief episode of loose stools following a visit to the Far East. He was treated with antibiotics and made a good recovery. There have only been a few case reports of *Salmonella* septic arthritis in a rheumatoid arthritis patient on anti–tumor necrosis factor treatment but none previously in association with certolizumab.

## Introduction

Rheumatoid arthritis (RA) is a chronic, autoimmune, systemic disease that principally affects the synovium. Its prevalence is about 1% worldwide.^1^ Women are affected 3 times more than men and onset is usually between 30 and 50 years.

Evidence has shown that early and aggressive management of RA with disease-modifying antirheumatic drugs (DMARDs) has proven beneficial in slowing disease progression.^2^ NICE guidance supports the multidisciplinary team approach with physiotherapy, occupational therapy, podiatry, and psychotherapy.^3^ Pharmacological treatment includes analgesics, steroids, and DMARDs. With the advent of biologics, a new era of DMARD therapy has introduced various drugs such as anti–tumor necrosis factor (anti-TNF), anti-CD 20, anti–interleukin (IL)-6,and anti-IL-1. We currently have five anti-TNF drugs in the market to treat RA. These are infliximab, adalimumab, etanercept, certolizumab, and golimumab.

Anti-TNF drugs have a relatively good safety profile but are associated with several adverse events, which include the risk of malignancy, demyelinating disorders, lupus-like syndrome, congestive heart failure, and opportunistic infections.^4^ Patients with severe RA are prone to infections. We present a case of *Salmonella* septic arthritis in an RA patient treated with certolizumab.

## Case Report

A 43-year-old British Indian business manager was treated for active RA. Over 3 years he had received sulfasalazine, hydroxychloroquine, and methotrexate (MTX). Due to inefficacy and adverse effects, the DMARDs were stopped and certolizumab was commenced according to NICE guidelines with ongoing high disease activity. Within 1 month of commencing certolizumab, he traveled to Hong Kong during which he had a brief episode of gastroenteritis with fever. This was a self-limiting illness with spontaneous recovery within 2 days. Fifteen days later, he presented to our Rheumatology Department for a routine clinic visit. Clinical examination revealed typical rheumatoid changes with restricted range of movements of MCP, wrist, and elbow joints. His inflammatory markers were not significantly elevated in keeping with good RA disease control.

A musculoskeletal ultrasound examination performed at this clinic attendance, which did not reveal significant disease activity at the MCP, PIP, or wrist joints. However, in contrast, an ultrasound (US) examination of his left elbow revealed a moderated degree of synovial thickening a small amount of fluid, and positive power Doppler suggested an active inflammatory process. Clinical examination showed no joint tenderness, mild swelling, and restricted range of movement (extension limited to 150°), which was unchanged from previous clinic attendances. Given the monoarticular activity revealed on US examination, a decision was taken to aspirate this joint under US guidance despite the lack of clinical features of infection. His joint aspirate showed pus cells, macrophages, and neutrophils. Culture grew *Salmonella enteritidis* species sensitive to amoxicillin and ceftriaxone. As he was once again out of the country, he was treated with oral ciprofloxacin for a week and reviewed on his return to the United Kingdom. As the elbow effusion persisted, he was admitted for intravenous co-amoxiclav treatment. A magnetic resonance imaging (MRI) scan of the left elbow showed erosions, effusion, synovitis, but no osteomyelitis. His blood culture showed *Salmonella enteritidis*, and stool cultures were negative. Repeat aspirate showed no organisms within the joint fluid. He was discharged home a week later with a 2-week course of oral co-amoxiclav. On recent review he remains well with no recurrence of elbow effusion.

## Discussion

A population-based study by Doran et al suggested that patients with RA were at increased risk of developing infections compared with non-RA subjects. This may be due to immunomodulatory effects of RA or due to agents with immunosuppressive effects used in its treatment.^5^

In further support of the notion of increased infection risk in patients with RA is the finding that up to 40% of patients with septic arthritis have RA.^6^ The annual incidence of septic arthritis in the general population is 2 to 5 per 100 000, and patients with preexisting RA were at an increased risk of septic arthritis (odds ratio = 4.0, 95% confidence interval = 1.9-8.3).^7^ One study suggested the annual frequency of septic arthritis to be 0.2% among patients with RA.^8^

Septic arthritis in patients with RA was initially described by Bywaters^9^ and by Kellgren and colleagues.^10^ Typically, septic arthritis presents with acute onset of severe joint pain, with very limited range of movements, accompanied by swelling and erythema at the affected joint. There are also systemic manifestations of infection including fever, elevated white blood cell count, and increased erythrocyte sedimentation rate. However, with RA the onset is more insidious and is mistaken for a flare of RA. The presentation may be with either a monoarticular or a polyarticular distribution. Polyarticular pattern may be seen in 10% to 20% of cases. The knee joint is involved in about 50% of cases. The presentation may be more subtle in patients on immunosuppressants (glucocorticoids, disease modifying drugs, and biologics).

Biologics such as adalimumab, infliximab, and etanercept have been in the scene for more than 10 years and have proven efficacy in RA. Certolizumab is a pegylated human monoclonal anti-TNF-α antibody and is one of the latest anti-TNF in the market. The RAPID 2 study showed that certolizumab pegol plus MTX was more efficacious than placebo plus MTX. The certolizumab combination rapidly and significantly improved clinical features, physical function, and inhibited radiographic progression.^11^

The anti-TNF drugs are associated with unusual and atypical viral and fungal infections, tuberculosis, and bacterial infections. The meta-analysis of randomized clinical trials, the German and Spanish registries, and a re-analysis of BSR Biologics Register (BSRBR) data suggest that TNF inhibitor use is associated with an approximate doubling of risk of serious infection, particularly early on in the course of therapy.^12-14^ The RAPID study showed 7% serious infections. Two cases of tuberculosis were reported, but there were no opportunistic infections.^11^

BSRBR data showed that patients on anti-TNF therapy were twice as likely to develop septic arthritis as controls (adjusted hazard ratio [HR] = 2.0, 95% confidence interval [CI] = 1.2-3.7). This risk was greatest during the first year of therapy, peaking at around 10 months after commencement. Among the responsible organisms found in 41% of cases, *Staphylococcus* was the most common organism in both cohorts (DMARD 55%; anti-TNF 50%), and several intracellular pathogens (including *Listeria* and *Salmonella*) were also reported with the anti-TNF cohort.

*Salmonella* are mobile gram-negative facultative intracellular anaerobes that cause a wide spectrum of disease ranging from gastroenteritis, enteric fever, and focal and disseminated infection. According to the World Health Organization, over 16 million people worldwide are infected with typhoid fever each year, with 500 000 to 600 000 fatal cases. Worldwide estimates of nontyphoid *Salmonella* range from 200 million to 1.3 billion, with an estimated death toll of 3 million each year.^15^ In the United States, the reported 2008 incidence was 16.2 cases per 100 000.^16^ Additionally, an estimated 500 people are infected with typhoid *Salmonella* annually. Most cases of documented typhoid disease are related to foreign travel to developing nations such as India (30%), Pakistan (13%), Mexico (12%), Bangladesh (8%), Philippines (8%), and Haiti (5%).^17^

*Salmonella* can be spread through contaminated raw eggs, in unpasteurized milk, and in undercooked meat. It rarely causes primary infection in the musculoskeletal system except in special circumstances such as following instrumentation, traumatic injury to the joint, or arthrocentesis. The risk of *Salmonella* septic arthritis is associated with infancy, elderly, or with diseases such as sickle cell anemia,^18-21^ acute lymphoblastic leukemia,^22,23^ thalassaemia,^24,25^ idiopathic thrombocytopenic purpura,^26^ Crohn’s disease,^27^ systemic lupus erythematosus,^28,29^ HIV,^30^ RA, and diabetes mellitus,^31^ or in special circumstances such as prosthetic joint,^32-36^ transplant,^37^ or avascular necrosis. It can also cause osteomyelitis septic arthritis or postinfectious reactive arthritis.^38^

BSRBR reported that the risk of septic arthritis with specific anti-TNF were etanercept HR 2.3 (CI = 1.2-4.4), infliximab HR 1.6 (CI = 0.8-3.2), and adalimumab HR 1.8 (CI = 1.0-3.5). Five cases of intracellular infection (2 *Listeria*, 3 *Salmonella*) and 11 cases of Streptococcal SA (including 4 *Streptococcus pyogenes*) were reported (all in the anti-TNF cohort). Katsarolis et al reported a case of septic arthritis due to *Salmonella enteritidis* in a patient on infliximab for RA.^39^ Antimicrobial chemotherapy with surgical intervention was necessary for eradication of the infection.

Wallis et al^40^ have identified 11 cases of anti-TNF-associated *Salmonella* infection (7 cases with infliximab and 4 cases with etanercept) from the FDA’s AERS. Two cases of *Salmonella* septic arthritis have been reported with etanercept and a further 2 cases have been reported with infliximab.^39,41^

Three cases of *Salmonella* septicemia have been reported with anti-TNF therapy.^42^ Fu et al reported a case of disseminated *Salmonella typhimurium* infection in a psoriatic arthritis patient with infliximab treatment.^43^ A case of disseminated *Salmonella paratyphi* infection in a RA patient treated with infliximab was reported.^44^ Netea et al hypothesized that TNF neutralization with monoclonal antibodies may result in a decreased production of interferon (IFN)-γ, subsequently leading to a defective cellular immune response and decreased expression of Toll-like receptor 4. They concluded that as recognition of microorganisms by TLR-4 and activation of phagocytes by IFN-γ are crucial mechanisms for the defence against intracellular pathogens, their inhibition by anti-TNF leads to severe complication with *Salmonella* infections.^45^

Quick diagnosis is essential to prevent joint destruction. Urgent aspiration of the affected joints is required, followed by antibiotic therapy. Because of the subtle presentation with RA and presence of some “sterile” joints, there is often a delay in the diagnosis of the septic arthritis in these patients. Blackburn et al^46^ reported an average delay in diagnosis of 13.7 days. Radiologic investigations such as bone and gallium scans are often not helpful at the onset in distinguishing between infectious arthritis and inflammatory arthritis in patients with underlying inflammatory arthritis.^47^

Grassi suggested that sonographic examination should be directed to the site of clinical symptoms, or where abnormalities or confusing findings are present on other imaging studies. In comparison with radiography, US has the potential advantage of depicting tendon lesions, enthesitis, fluid collection, synovial proliferation, cartilage damage, and even minimal interruptions of the cortical bone profile that are frequently missed by conventional radiography because of their size and localization.^48^

In the same study, sonographic erosions that were not visible on radiography corresponded by site to MRI bone abnormalities. Hau and coworkers showed that ultrasonography provides better results than clinical examination alone.^49^ Kraan and colleagues showed that ultrasonography and MRI both can detect subclinical synovitis with corroborative macroscopic and microscopic data from arthroscopy in clinically normal knees of patients with RA.^50^ Karim and coworkers compared ultrasonography of the knee with the “gold standard” of arthroscopy, as well as clinical examination, to validate ultrasonographic images in terms of accurate representation of the pathology present in the joint.^51^ They concluded that ultrasonography was valid and reproducible, as well as superior to clinical examination for detecting knee synovitis.

Makkuni et al emphasized on hygiene advice in patients on anti-TNF therapy. They felt counseling was very important as there were particularly no vaccines against *Salmonella paratyphi*.^41^ Inflammatory diseases and biologics both increase risk of unusual infections. Thus, one needs to be vigilant as patients receiving biological therapy may have an insidious course to any infective process and use US in the assessment.

## References

[1] Lawrence JS Prevalence of rheumatoid arthritis. Ann Rheum Dis. 1961;20:11-17. 13759692 10.1136/ard.20.1.11 PMC1007178

[2] Luqmani R Hennell S Estrach C; BSR and BHPR Standards, Guidelines and Audit Working Group. British Society for Rheumatology and British Health Professionals in Rheumatology guideline for the management of rheumatoid arthritis (the first 2 years). Rheumatology (Oxford). 2009;48:436-439. 19174570 10.1093/rheumatology/ken450a

[3] National Institute for Health and Clinical Excellence. Rheumatoid Arthritis: National Clinical Guideline for Management and Treatment in Adults (Clinical Guidelines, No. 79). London, England: Royal College of Physicians; 2 2009. 21413195

[4] Furst DE Breedveld FC Kalden JR Updated consensus statement on biological agents for the treatment of rheumatoid arthritis and other rheumatic diseases (May 2002). Ann Rheum Dis. 2002;61(suppl II):ii2-ii7. 12379612 10.1136/ard.61.suppl_2.ii2 PMC1766714

[5] Doran MF Crowson CS Pond GR O’Fallon WM Gabriel SE Frequency of infection in patients with rheumatoid arthritis compared with controls: a population-based study. Arthritis Rheum. 2002;46:2287-2293. 12355475 10.1002/art.10524

[6] Goldenberg DL Infectious arthritis complicating rheumatoid arthritis and other chronic rheumatic disorders. Arthritis Rheum. 1989;32:496-502. 2650687 10.1002/anr.1780320422

[7] Kaandorp CJ Van Schaardenburg D Krijnen P Habbema JD van de Laar MA Risk factors for septic arthritis in patients with joint disease. A prospective study. Arthritis Rheum. 1995;38:1819-1825. 8849354 10.1002/art.1780381215

[8] Ostensson A Geborek P Septic arthritis as a non-surgical complication in rheumatoid arthritis: relation to disease severity and therapy. Br J Rheumatol. 1991;30:35-38. 1991215 10.1093/rheumatology/30.1.35

[9] Bywaters EG Fistulous rheumatism; a manifestation of rheumatoid arthritis. Ann Rheum Dis. 1953;12:114-121. 13058279 10.1136/ard.12.2.114 PMC1030466

[10] Kellgren JH Ball J Fairbrother RW Barnes KL Suppurative arthritis complicating rheumatoid arthritis. Br Med J. 1958;1:1193-1199. 13536456 10.1136/bmj.1.5081.1193 PMC2028760

[11] Smolen J Landewé RB Mease P Efficacy and safety of certolizumab pegol plus methotrexate in active rheumatoid arthritis: the RAPID 2 study. A randomized controlled trial. Ann Rheum Dis. 2009;68:797-804. 19015207 10.1136/ard.2008.101659 PMC2674556

[12] Bongartz T Sutton AJ Sweeting MJ Buchan I Matteson EL Montori V Anti-TNF antibody therapy in rheumatoid arthritis and the risk of serious infections and malignancies: systematic review and meta-analysis of rare harmful effects in randomized controlled trials. JAMA. 2006;295:2275-2285. 16705109 10.1001/jama.295.19.2275

[13] Listing J Strangfeld A Kary S Infections in patients with rheumatoid arthritis treated with biologic agents. Arthritis Rheum. 2005;52:3403-3412. 16255017 10.1002/art.21386

[14] Dixon WG Watson K Lunt M Hyrich KL Silman AJ Symmons DP; British Society for Rheumatology Biologics Register. Rates of serious infection, including site-specific and bacterial intracellular infection, in rheumatoid arthritis patients receiving anti-tumor necrosis factor therapy: results from the British Society for Rheumatology Biologics Register. Arthritis Rheum. 2006;54:2368-2376. 16868999 10.1002/art.21978

[15] Coburn B Grassl GA Finlay BB Salmonella, the host and disease: a brief review. Immunol Cell Biol. 2007;85:112-118. 17146467 10.1038/sj.icb.7100007

[16] Centers for Disease Control and Prevention. Preliminary FoodNet Data on the incidence of infection with pathogens transmitted commonly through food—10 States, 2008. MMWR Morb Mortal Wkly Rep. 2009;58:333-337. 19357633

[17] Linam WM Gerber MA Changing epidemiology and prevention of Salmonella infections. Pediatr Infect Dis J. 2007;26:747-748. 17848890 10.1097/INF.0b013e3181376abc

[18] Hernigou P Daltro G Flouzat-Lachaniette CH Roussignol X Poignard A Septic arthritis in adults with sickle cell disease often is associated with osteomyelitis or osteonecrosis. Clin Orthop Relat Res. 2010;468:1676-1681. 19885711 10.1007/s11999-009-1149-3 PMC2865595

[19] Akakpo-Numado GK Gnassingbe K Songne B Amadou A Tekou H Hip septic arthritis in young children with sickle-cell disease. Rev Chir Orthop Reparatrice Appar Mot. 2008;94:58-63. 18342031 10.1016/j.rco.2007.09.004

[20] Anand AJ Glatt AE Salmonella osteomyelitis and arthritis in sickle cell disease. Semin Arthritis Rheum. 1994;24:211-221. 7899877 10.1016/0049-0172(94)90076-0

[21] al-Salem AH Ahmed HA Qaisaruddin S al-Jam'a A Elbashier AM al-Dabbous I Osteomyelitis and septic arthritis in sickle cell disease in the eastern province of Saudi Arabia. Int Orthop. 1992;16:398-402. 1473897 10.1007/BF00189627

[22] Xanthaki A Melita-Manolis H Toutouza-Yotsa M Voyiatzakis E Treloyianni X Komninou Z Septic arthritis due to Salmonella in a patient with acute lymphatic leukemia. Acta Microbiol Hellenica. 1991;36:471-478.

[23] Yang WC Huang YC Tsai MH Chiu CH Jaing TH Septic arthritis involving multiple joints in a girl with acute lymphoblastic leukemia at diagnosis. Pediatr Neonatol. 2009;50:33-35. 19326836 10.1016/S1875-9572(09)60027-9

[24] Rayan F Mukundan C Shukla DD A case of relapsing Salmonella osteomyelitis in a thalassaemia trait patient. J Orthop Traumatol. 2009;10:31-33. 19384633 10.1007/s10195-008-0033-3 PMC2657351

[25] Behera B Mathur P Farooque K Sharma V Bhardwaj N Thakur YK Salmonella enterica enteritidis arthritis following trauma in a child with thalassemia major. Indian J Pediatr. 2010;77:807-808. 20589479 10.1007/s12098-010-0102-5

[26] Kanra G Seçmeer G Toyran M Cengiz AB Değertekin Y Kara A Salmonella septic arthritis in a patient with acute idiopathic thrombocytopenic purpura treated with steroid. Turk J Pediatr. 2000;42:151-154. 10936983

[27] Rim JY Tenorio AR Salmonella septic arthritis in a patient with Crohn’s disease on infliximab. Inflamm Bowel Dis. 2010;16:545-547. 19637386 10.1002/ibd.21033

[28] Gerona JG Navarra SV Salmonella infections in patients with systemic lupus erythematosus: a case series. Int J Rheum Dis. 2009;12:319-323. 20374369 10.1111/j.1756-185X.2009.01440.x

[29] Maderazo EC Navarra SV Salmonella arthritis in a Filipino patient with lupus nephritis: case report. Int J Rheum Dis. Conference: 14th Congress of Asia Pacific League of Associations for Rheumatology, APLAR 2010 Hong Kong, Hong Kong; July 2010.

[30] Garcia Forcada IL Calmet Garcia J Gine I Goma J Septic arthritis due to Salmonella in two HIV-positive hemophiliacs. Rev Ortop Traumatol. 1999;43:365-367.

[31] Suwannaroj S Mootsikapun P Vipulakorn K Nanagara R Salmonella group D septic arthritis and necrotizing fasciitis in a patient with rheumatoid arthritis and diabetes mellitus. J Clin Rheumatol. 2001;7:83-85. 17039101 10.1097/00124743-200104000-00006

[32] Miron D Zuker M Lev-El A Salmonella prosthetic knee septic arthritis successful retention of the prosthesis with prolonged suppressive therapy. Harefuah. 2006;145:261-263. 16642625

[33] Day LJ Qayyum QJ Kauffman CA Salmonella prosthetic joint septic arthritis. Clin Microbiol Infect. 2002;8:427-430. 12199853 10.1046/j.1469-0691.2002.00466.x

[34] Madan S Abbas D Jowett RL Mounce K Salmonella enteritidis infection in total knee replacement. Rheumatology (Oxford). 2001;40:112-113. 11157155 10.1093/rheumatology/40.1.112

[35] Kobayashi H Hall GS Tuohy MJ Knothe U Procop GW Bauer TW Bilateral periprosthetic joint infection caused by Salmonella enterica serotype Enteritidis, and identification of Salmonella sp using molecular techniques. Int J Infect Dis. 2009;13:e463-e466. 19269872 10.1016/j.ijid.2008.12.015

[36] Boland F Kaushik P Udo EE Rotimi VO Malaviya AN Salmonella septic arthritis complicating rheumatoid arthritis in a patient with total knee replacement. Med Princip Pract. 1999;8:245-250.

[37] Martin-Santos JM Alonso-Pulpon L Pradas G Septic arthritis by Salmonella enteritidis after heart transplantation. J Heart Transplant. 1987;6:177-179. 3309221

[38] Simonet ML Enterobacteria in reactive arthritis: Yersinia, Shigella, and Salmonella. Rev Rhum Engl Ed. 1999;66(1 suppl):14S-19S. 10063518

[39] Katsarolis I Tsiodras S Panagopoulos P Septic arthritis due to Salmonella enteritidis associated with infliximab use. Scand J Infect Dis. 2005;37:304-306. 15804667 10.1080/00365540410021171

[40] Wallis RS Broder MS Wong JY Hanson ME Beenhouwer DO Granulomatous infectious diseases associated with tumor necrosis factor antagonists. Clin Infect Dis. 2004;38:1261-1265. 15127338 10.1086/383317

[41] Makkuni D Kent R Watts R Clunie G Two cases of serious food-borne infection in patients treated with anti-TNF-alpha. Are we doing enough to reduce the risk? Rheumatology. 2006;45:237-238. 16377730 10.1093/rheumatology/kei123

[42] Rijkeboer A Voskuyl A Van Agtmael M Fatal Salmonella enteritidis septicaemia in a rheumatoid arthritis patient treated with a TNF-alpha antagonist. Scand J Infect Dis. 2007;39:80-83. 17366020 10.1080/00365540600786549

[43] Fu A Bertouch JV McNeil HP Disseminated Salmonella typhimurium infection secondary to infliximab treatment. Arthritis Rheum. 2004;9:3049-3060. 15457476 10.1002/art.20639

[44] Bassetti M Nicco E Delfino E Viscoli C Disseminated Salmonella paratyphi infection in a rheumatoid arthritis patient treated with infliximab. Clin Microbiol Infect. 2009;16:84-85. 19723134 10.1111/j.1469-0691.2009.02961.x

[45] Netea MG Radstake T Joosten LA van der Meer JW Barrera P Kullberg BJ Salmonella septicemia in rheumatoid arthritis patients receiving anti-tumor necrosis factor therapy: association with decreased interferon-gamma production and Toll-like receptor 4 expression. Arthritis Rheum. 2003;48:1853-1857. 12847679 10.1002/art.11151

[46] Blackburn WD Jr Dunn TL Alarcon GS Infection versus disease activity in rheumatoid arthritis: eight years’ experience. South Med J. 1986;79:1238-1241. 3764520 10.1097/00007611-198610000-00011

[47] Kherani RB Shojania K Septic arthritis in patients with pre-existing inflammatory arthritis. CMAJ. 2007;176:1605-1608. 17515588 10.1503/cmaj.050258 PMC1867838

[48] Grassi W Clinical evaluation versus ultrasonography: who is the winner? J Rheumatol. 2003;30:908-909. 12734880

[49] Hau M Schultz H Tony HP Evaluation of pannus and vascularization of the metacarpophalangeal and proximal interphalangeal joints in rheumatoid arthritis by high-resolution ultrasound (multidimensional linear array). Arthritis Rheum. 1999;42:2303-2308. 10555024 10.1002/1529-0131(199911)42:11<2303::AID-ANR7>3.0.CO;2-4

[50] Kraan MC Versendaal H Jonker M Asymptomatic synovitis precedes clinically manifest arthritis. Arthritis Rheum. 1998;41:1481-1488. 9704648 10.1002/1529-0131(199808)41:8<1481::AID-ART19>3.0.CO;2-O

[51] Karim Z Wakefield RJ Quinn M Validation and reproducibility of ultrasonography in the detection of synovitis in the knee: a comparison with arthroscopy and clinical examination. Arthritis Rheum. 2004;50:387-394. 14872480 10.1002/art.20054

